# An Assessment of the Impact of SARS-CoV-2 on a Level 1 Trauma Center Including Subgroup Analysis of Orthopedic Injuries and Mechanism of Injury

**DOI:** 10.7759/cureus.20954

**Published:** 2022-01-05

**Authors:** Austin Moore, Amy Singleton, Logan Hiatt, Richard Miller, Seth Phillips, John J Leskovan

**Affiliations:** 1 Orthopaedic Surgery, Mercy Health St. Vincent Medical Center, Toledo, USA; 2 Department of Trauma Surgery, Mercy Health St. Vincent Medical Center, Toledo, USA

**Keywords:** surgery, orthopedics, trauma, epidemiology, fracture, sars-cov-2, covid

## Abstract

Background and objective

There is a paucity of medical literature describing the preparedness of hospital institutions to withstand the population effects of a pandemic. Severe acute respiratory syndrome coronavirus 2 (SARS-CoV-2), the virus that causes coronavirus disease 2019 (COVID-19), has had a global impact on all facets of medicine, which has ultimately affected the medical community in a significant manner. Furthermore, there is a scarcity of research regarding the effects of COVID-19 on trauma and acute care surgery injury and admission rates. We conducted this study to examine the effects of the COVID-19 pandemic on both pediatric and adult trauma admissions, injury types, and mechanisms of injury.

Materials and methods

Data from the Trauma Registry was extracted for all adult (>15 years) and pediatric (<15 years) patients who consulted trauma surgery, acute care surgery, or orthopedic surgery at our center in the year immediately prior to the pandemic (March 1, 2019-February 29, 2020) and during the COVID-19 pandemic (March 1, 2020-February 28, 2021). Patient demographics, cause of injury, injury type and mechanism, and procedures performed were recorded.

Results

We documented a 4.2% increase in adult encounters compared to the preceding year. There was a significant difference in the distribution of mechanism of injury of adult patients between the two time periods, with the most changes seen in motor-vehicle auto, gunshot, and other vehicle injuries. However, no significant difference was seen in trauma type or intent (assault, self-inflicted, unintentional). Pediatric encounters increased by 6.4% during the COVID-19 pandemic compared to the pre-COVID-19 period. Overall, there was no detectable association between the distribution of encounters by the mechanism of injury and the time period for pediatric encounters.

Conclusion

This retrospective review of trauma encounters through both pre-COVID-19 and COVID-19 periods outlines the differences in factors such as demographics, injury mechanisms, and injury types between the two time periods. Overall, we expected a decrease in orthopedic-related trauma admissions during the COVID-19 pandemic; however, there was actually an increase of 4.1% in adult encounters and that of 6.4% in pediatric encounters. Our study lays out possible trends in injury patterns that can be correlated with the COVID-19 pandemic and the lockdown period. This information is useful for the healthcare system in that it demonstrates that resources should not be cut down or removed from surgical specialties. At level I facilities, resources need to be allocated for and continued to be provided to emergency rooms and operative services, including supplies and staffing. These departments need to be well-equipped to handle an increased number of trauma patients.

## Introduction

There is a scarcity of data in the medical literature about the preparedness of healthcare systems and hospitals to deal with the population effects of a pandemic. The coronavirus disease 2019 (COVID-19) pandemic has had a global impact on all facets of medicine, resulting in significant stress and challenges in the medical community. Furthermore, there is a scarcity of research regarding the effects of COVID-19 on trauma and acute care surgery injury and admission rates. Hospitals can better prepare for future pandemics and dramatic shifts in healthcare needs if the authorities are well-equipped with the knowledge related to average changes in injury incidence and admission rates that a pandemic or a similar situation brings about.

With the implementation of social distancing and quarantine measures to better control the spread of COVID-19, it could be surmised that a hospital’s trauma surgery census would have decreased overall as many people have been prevented from engaging in high-risk activities that usually cause traumatic injuries. To our knowledge, there is no study in the literature analyzing the effect of COVID-19 on traumatic injuries or admissions in northwest Ohio so far.

Multiple studies have analyzed the incidence of injuries, trauma surgery, and orthopedic surgery during the COVID-19 pandemic. A systematic review performed in Singapore observed a decrease in elective, traumatic, and outpatient orthopedic surgeries by 80%, 47%, and 63%, respectively, since the beginning of COVID-19 [1]. They found an overall proportional increase in domestic injuries and poly-traumas, which they attributed to patients with minor injuries potentially avoiding treatment to reduce the risk of COVID-19 exposure. One study out of Michigan observed an overall decrease of 45% in orthopedic trauma encounters at a level II trauma center after the shelter-in-place order was established [2]. Other studies have shown a trend of decreased pediatric and adult trauma and orthopedic admissions since the onset of the COVID-19 pandemic; however, none of these studies have reported on the incidence of injuries or changes in trauma admissions in northwest Ohio region [3-9].

Our institution is an urban, level 1 trauma center for adults and level 2 for children with approximately 60,000 emergency room visits and 2,400 trauma admissions annually. The institution is home to multiple graduate medical education programs and is a well-established, longstanding tertiary care center in the Midwestern United States. The purpose of this study was to describe the effects of the COVID-19 pandemic on both pediatric and adult trauma admissions, injury types, and mechanisms of injury.

## Materials and methods

Data from the Trauma Registry was extracted for all adult (>15 years) and pediatric (<15 years) patients who consulted our institution's trauma surgery, acute care surgery, or orthopedic surgery in the year immediately prior to the pandemic (March 1, 2019-February 29, 2020) and during the COVID-19 pandemic (March 1, 2020-February 28, 2021).

The primary analyses involved univariate comparisons of two independent groups of patients based on the time period: pre-COVID-19 and during COVID-19. To demonstrate that the study has adequate power to detect meaningful differences, we calculated the sample size needed to detect a 15% difference between groups or small effect size, as follows: with 5% type I error, 80% power, and a two-tailed test of binomial proportions, a 15% difference was detectable with n=385 per time period (e.g., 50% with the outcome of interest in one time period vs. 65% in the other time period). For continuous outcomes, an adequate number of patients (>385) per time period was available to detect small effect sizes (e.g., the mean difference between two time periods of 0.2 with a standard deviation of 1).

Categorical variables and outcomes are presented as frequency counts, percentages, and 95% confidence intervals, and compared between time periods (pre-COVID-19 vs. COVID-19) using Chi-square or Fisher’s exact tests. Continuous variables and outcomes are presented as means ± standard deviations, and 95% confidence intervals (or medians and interquartile ranges if the data are not distributed normally) and compared between time periods (pre-COVID-19 vs. COVID-19) with Student's t-tests or Mann-Whitney-Wilcoxon tests. All p-values are two-tailed.

## Results

Adult patients

**Figure 1:**
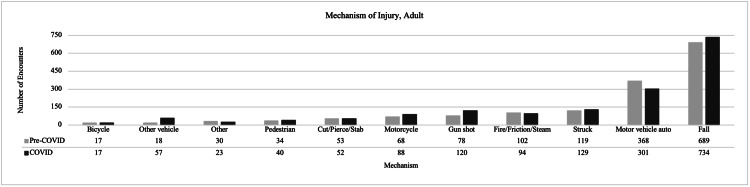
Mechanism of injury in adult patient encounters during pre-COVID-19 vs. COVID-19 COVID-19: coronavirus disease 2019

There were more adult encounters during the COVID-19 pandemic compared to the preceding year, representing a 4.1% increase: 1,631 during pre-COVID-19 vs. 1,698 during COVID-19. For adult encounters, there was a significant difference in the distribution of patients by race; there was a higher proportion of Black/African American encounters during COVID-19 compared to the pre-COVID-19 period. The demographic details are shown in Table 1. There was a significant difference in the distribution of mechanism of injury of adult patients between the time periods, with the most changes seen in motor-vehicle auto, gunshot, and other-vehicle injuries that include golf cart, plane, jet ski, ATV, electric scooter or bike, snowmobile, or boat (Figure 1). During COVID-19, there was a higher percentage of encounters from injuries due to gunshots (7.1% of 1,698 encounters during COVID-19 vs. 4.8% of 1,631 pre-COVID-19 encounters), fewer encounters from motor-vehicle auto accidents (17.9% vs. 22.8%), and more encounters due to other vehicles (3.4% vs. 1.1%), as seen in Figure 1. We observed higher rates of open reduction internal fixations (ORIFs) (13.8% vs. 10.9%) performed during COVID-19, but fewer external fixation placements (0.3% vs. 0.9%). Similar numbers of open reduction, closed reduction, exploratory laparotomy, and incision and drainage were encountered in the pre-COVID-19 period and during COVID-19. There was no significant difference seen in trauma type or intent (assault, self-inflicted, unintentional). There were no detectable differences in age, sex, other mechanisms of injury, other procedures, alcohol, ICU length of stay (LOS), or Injury Severity Score (ISS). Further details can be found in Table 2.

**Table 1:** Demographics of adult and pediatric patients in the pre-COVID-19 and COVID-19 periods *Significant p-value (<0.01) COVID-19: coronavirus disease 2019

	Pre-COVID-19	COVID-19	P-value
Arrival date range	03/2019–02/2020	03/2020–02/2021	
Adult patients			
Number of patients	1,631	1,698	
Age in years, mean ± SD	53.5 ± 22.0	52.1 ± 21.6	0.06
Race, n (%)			<0.001*
White	1,233 (75.6)	1,256 (74.2)	0.33
Black/African American	273 (16.8)	350 (20.7)	<0.01*
Other	124 (7.6)	87 (5.1)	<0.01*
Sex, n (%)			0.6
Female	591 (36.2)	630 (37.1)	
Male	1,040 (63.8)	1,068 (62.9)	
Pediatric patients			
Number of patients	171	182	
Age in years, mean ± SD	5.7 ± 4.5	6.7 ± 4.7	0.06
Race, n (%)			0.17
White	110 (65.1)	128 (70.3)	0.29
Black/African American	32 (18.9)	37 (20.3)	0.74
Other	27 (16.0)	17 (9.3)	0.06
Sex, n (%)			0.03
Female	74 (43.3)	58 (31.9)	
Male	97 (56.7)	124 (68.1)	

**Table 2:** Statistics for adult patients Pre-COVID-19 and COVID-19 results are compared using Chi-square tests (or Fisher’s Exact test) for categorical variables, Student's t-test for age, and Mann-Whitney-Wilcoxon test for ICU days, total LOS, and ISS COVID-19: coronavirus disease 2019; EtOH: ethanol; FFP: fresh frozen plasma; IQR: interquartile range; ISS: Injury Severity Score; LOS: length of stay; SD: standard deviation

Adult patients (age: ≥15 years)			
	Pre-COVID-19 (3/1/2019–2/29/2020)	COVID-19 (3/1/2020–2/28/2021)	P-value
Number of patients	1,631	1,698	
Age in years, mean ± SD	53.5 ± 22.0	52.1 ± 21.6	0.06
Race, n (% of N)			<0.001
White	1,233 (75.6)	1,256 (74.2)	0.33
Black/African American	273 (16.8)	350 (20.7)	<0.01
Other	124 (7.6)	87 (5.1)	<0.01
Sex, n (% of N)			0.6
Female	591 (36.2)	630 (37.1)	
Male	1,040 (63.8)	1,068 (62.9)	
Mechanism of injury, n (% of N)			<0.001
Bicycle	17 (1.1)	17 (1.0)	0.9
Cut/pierce/stab	53 (3.3)	52 (3.1)	0.75
Fall	689 (42.8)	734 (43.7)	0.6
Fire/friction/steam	102 (6.3)	94 (5.6)	0.37
Gunshot	78 (4.8)	120 (7.1)	0.01
Hanging/suffocation	9 (0.6)	6 (0.4)	0.39
Inhalation	11 (0.7)	9 (0.5)	0.59
Machinery	13 (0.8)	8 (0.5)	0.23
Motor-vehicle auto	368 (22.8)	301 (17.9)	<0.001
Motorcycle	68 (4.2)	88 (5.2)	0.17
Near-drowning	2 (0.1)	3 (0.2)	1.0
Other	30 (1.9)	23 (1.4)	0.26
Other vehicles	18 (1.1)	57 (3.4)	<0.001
Pedestrian	34 (2.1)	40 (2.4)	0.6
Struck	119 (7.4)	129 (7.6)	0.75
Intent, n (% of N)			0.22
Assault	152 (9.5)	187 (11.3)	0.11
Self-inflicted	27 (1.7)	26 (1.6)	0.78
Unintentional	1,417 (88.7)	1,445 (86.9)	0.12
War	1 (0.1)	4 (0.2)	
Work-related Injury, n (% of N)	109 (6.7)	93 (5.5)	0.15
Trauma type, n (% of N)			0.13
Asphyxia	6 (0.4)	5 (0.3)	0.71
Blunt	1,372 (84.1)	1,415 (83.3)	0.54
Burns/cold	108 (6.6)	105 (6.2)	0.61
Drowning	26 (1.6)	16 (0.9)	0.09
Penetrating	119 (7.3)	157 (9.3)	0.04
ED hypotension	156 (9.7)	174 (10.4)	0.49
Non-accidental trauma	15 (0.9)	11 (0.7)	0.37
Closed reduction	170 (10.4)	179 (10.5)	0.91
Oral intubation	252 (15.4)	310 (18.3)	0.03
Nasal intubation	4 (0.3)	1 (0.1)	0.21
Open reduction	9 (0.6)	10 (0.6)	0.89
Open reduction with internal fixation	178 (10.9)	235 (13.8)	0.01
Open reduction with external fixation	15 (0.9)	5 (0.3)	0.02
Amputation	6 (0.4)	11 (0.7)	0.26
Bone graft	1 (0.1)	1 (0.1)	1.0
Exploratory laparotomy	42 (2.6)	43 (2.5)	0.94
Fasciotomy	9 (0.6)	11 (0.7)	0.72
FFP transfusion	71 (4.4)	71 (4.2)	0.81
Graft	14 (0.9)	14 (0.8)	0.91
Hip replacement	21 (1.3)	22 (1.3)	0.98
Incision and drainage	8 (0.5)	3 (0.2)	0.11
Tracheostomy	30 (1.8)	31 (1.8)	0.98
Toxicology results (among those tested), n (% of N)			0.003
Tested, no positive result	321 (60.2)	302 (51.3)	
Positive for >1 drug	212 (39.7)	287 (48.7)	
EtOH above the legal limit (among those tested), n (% of N)			0.69
>0.08	250 (22.9)	271 (23.6)	
<0.08	844 (77.2)	879 (76.4)	
ICU LOS, days, median (IQR)	2 (1–4)	2 (1–5)	0.17
	n=607 with >1 days	n=548 with >1 days; n=5 with 0 days	
Total LOS, days, median (IQR)	2 (1–5)	2 (1–5)	0.01
Injury Severity Score (ISS), median (IQR)	9 (5–14)	9 (5–14)	

Pediatric patients

**Figure 2:**
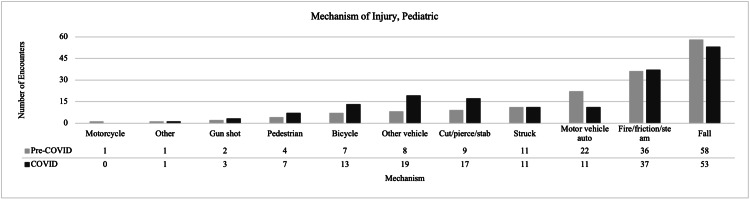
Mechanism of injury in pediatric patient encounters for pre-COVID-19 vs. COVID-19 COVID-19: coronavirus disease 2019

Pediatric encounters increased by 6.4% from 171 during the pre-COVID-19 period to 182 during COVID-19. There were more male pediatric encounters during COVID-19 compared to pre-COVID-19 (68.1% vs. 56.7%). The demographic details are shown in Table 1. During COVID-19, there was a higher percentage of encounters related to pedestrian (3.9% vs. 2.4%), bicycle (7.3% vs. 4.2%), other-vehicle (10.6% vs. 4.9%), and cut/pierce/stab (9.5% vs. 5.5%) injuries, as seen in Figure 2. However, we observed a lower proportion of motor-vehicle auto injuries (6.2% vs. 13.3%) and falls (29.6% vs. 35.2%) during COVID-19 compared to pre-COVID-19. Overall, there was no detectable association between the distribution of encounters by the mechanism of injury and time period. None of the statistical tests we performed for the mechanism of injury met our stringent p<0.01 criteria for multiple testing; however, there was some evidence with p<0.05 with regard to fewer MVAs during COVID-19 compared to pre-COVID-19 (6.2% vs. 13.3%). There were fewer non-accidental traumas in the COVID-19 period compared to pre-COVID-19 (5.0% vs. 17.0%). Small sample sizes for other variables of interest reduced our ability to detect any statistical differences. Further details can be found in Table 3.

**Table 3:** Statistics for pediatric patients Pre-COVID-19 and COVID-19 results are compared using Chi-square tests (or Fisher’s exact tests) for categorical variables, Student's t-test for age, and Mann-Whitney-Wilcoxon test for ICU days, total LOS, and ISS COVID-19: coronavirus disease 2019; EtOH: ethanol; FFP: fresh frozen plasma; IQR: interquartile range; ISS: Injury Severity Score; LOS: length of stay; SD: standard deviation

Pediatric patients (age: ≤15 years)			
	Pre-COVID-19 (3/1/2019–2/29/2020)	COVID-19 (3/1/2020–2/28/2021)	P-value
Number of patients	171	182	
Age in years, mean ± SD	5.7 ± 4.5	6.7 ± 4.7	0.06
Race, n (% of N)			0.17
White	110 (65.1)	128 (70.3)	0.29
Black/African American	32 (18.9)	37 (20.3)	0.74
Other	27 (16.0)	17 (9.3)	0.06
Sex, n (% of N)			0.03
Female	74 (43.3)	58 (31.9)	
Male	97 (56.7)	124 (68.1)	
Mechanism of injury, n (% of N)			0.25
Bicycle	7 (4.2)	13 (7.3)	0.23
Cut/pierce/stab	9 (5.5)	17 (9.5)	0.16
Fall	58 (35.2)	53 (29.6)	0.27
Fire/friction/steam	36 (21.8)	37 (20.7)	0.79
Gunshot	2 (1.2)	3 (1.7)	1.0
Hanging/suffocation	2 (1.2)	1 (0.6)	0.61
Inhalation	0 (0)	0 (0)	No test
Machinery	0 (0)	0 (0)	No test
Motor-vehicle auto	22 (13.3)	11 (6.2)	0.02
Motorcycle	1 (0.6)	0 (0)	No test
Near-drowning	4 (2.4)	6 (3.4)	0.75
Other	1 (0.6)	1 (0.6)	No test
Other vehicles	8 (4.9)	19 (10.6)	0.05
Pedestrian	4 (2.4)	7 (3.9)	0.43
Struck	11 (6.7)	11 (6.2)	0.84
Intent, n (% of N)			0.48
Assault	1 (0.6)	0 (0)	No test
Self-inflicted	0 (0)	0 (0)	No test
Unintentional	157 (99.4)	168 (100)	0.48
Work-related Injury	0 (0)	0 (0)	No test
Trauma type, n (% of N)			0.54
Asphyxia	1 (0.6)	0 (0)	No test
Blunt	125 (73.1)	129 (70.9)	0.64
Burns/cold	36 (21.1)	38 (20.9)	0.97
Drowning	6 (3.5)	7 (3.9)	0.87
Penetrating	3 (1.8)	8 (4.4)	0.15
ED hypotension	4 (2.7)	6 (3.8)	0.75
Non-accidental trauma	29 (17.0)	9 (5.0)	<0.001
Closed reduction	15 (8.8)	27 (14.8)	0.08
Oral intubation	8 (4.7)	11 (6.0)	0.57
Nasal intubation	0 (0)	1 (0.6)	No test
Open reduction	0 (0)	0 (0)	No test
Open reduction with internal fixation	9 (5.3)	9 (5.0)	0.89
Open reduction with external fixation	1 (0.6)	0 (0)	No test
Amputation	0 (0)	0 (0)	No test
Bone graft	0 (0)	0 (0)	No test
Exploratory laparotomy	2 (1.2)	1 (0.6)	0.61
Fasciotomy	0 (0)	0 (0)	No test
FFP transfusion	0 (0)	1 (0.6)	No test
Graft	2 (1.2)	0 (0)	0.23
Hip replacement	0 (0)	0 (0)	No test
Incision and drainage	0 (0)	0 (0)	No test
Tracheostomy	0 (0)	0 (0)	No test
Toxicology results (among those tested), n (% of N)			1.0
Tested, no positive result	6 (85.7)	15 (88.2)	
Positive for >1 drug	1 (14.3)	2 (11.8)	
EtOH above the legal limit (among those tested), n (% of N)			
>0.08	0 (0)	0 (0)	
<0.08	54 (100)	79 (100)	
ICU LOS, days, median (IQR)	1 (1–2)	1 (1–2)	0.44
	n=103 with >1 days; 68 missing	n=54 with >1 days; 129 missing	
Total LOS, days, median (IQR)	1 (1–2)	1 (1–2)	0.95
	n=170	n=166	
Injury Severity Score (ISS), median (IQR)	4 (1–9)	5 (1–9)	0.48

Injury type

**Figure 3:**
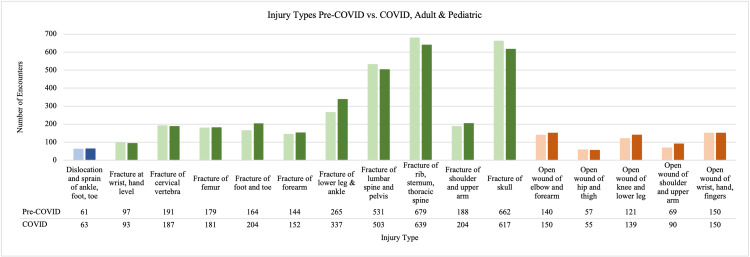
Injuries where the count was >52 for either pre-COVID-19 or COVID-19 (1 injury per week) Only orthopedic-related injuries are reported here. Pre-COVID-19 numbers are presented as the first bar followed by COVID-19 numbers as the second bar. Blue represents dislocation injuries, green fracture injuries, and orange stands for open wound injuries COVID-19: coronavirus disease 2019

For injury types in the combined adult and pediatric population, we looked at injuries that occurred at least on a weekly basis, with a count >52 for the time period (Figure 3). We excluded injuries that were not orthopedic-related and then compared the time frames. There were more fractures of the cervical vertebra, femur, foot and toe, forearm, lower leg and ankle, and shoulder and upper arm during COVID-19 compared to pre-COVID-19. During COVID-19, there were more open wounds of the elbow and forearm, knee and lower leg, and shoulder and upper arm. Conversely, there were fewer fractures of the lumbar spine and pelvis, skull, open wound of the hip and thigh, and an equal number of open wounds of the wrist, hand, and fingers during COVID-19 compared to pre-COVID-19.

## Discussion

This retrospective review of trauma encounters through both pre-COVID-19 and COVID-19 time periods outlines the differences in demographics, injury mechanisms, and injury types between both time periods. Overall, we expected a decrease in orthopedic-related trauma admissions during the COVID-19 pandemic vs. the preceding year, but this was not seen in our data. There was in fact an increase of 4.1% in adult encounters, and that of 6.4% in pediatric encounters. This is in direct contrast with articles that have shown dramatic decreases of up to 47% in orthopedic trauma admissions [1,2]. There were more pediatric encounters during COVID-19, but there was no significant change seen in the overall distribution of mechanism of injury (Figure 2), intent, or trauma type (Table 2). It was thought that due to limited school time, summer camps, and activities, the pediatric encounters would be lower in terms of numbers, mechanism of injury, and trauma type.

There were significant differences in the distribution of adult patients by race (p<0.001) but not by sex (p=0.6) or age (p=0.06). Interestingly, a higher change in the proportion of Black/African Americans (20.7% vs. 16.8%) was seen during COVID-19 than pre-COVID-19 compared to the white population (75.6% vs. 74.2%). The population percentages do appear to align with the community ethnic makeup reported in the 2019 Community Health Needs Assessment Report; 68.8% Caucasian and 20.1% Black; but the change in percentage was almost 3x different (3.9% vs. 1.4%) [10]. The larger increase seen during COVID-19 may be due to COVID-19 disproportionately affecting the African American community. It has been shown that African American mortality rate is twice that of whites and Asians, leading to increased hospital encounters of this racial group [11]. Similarly, this trend has also been noticed in terms of traumatic injuries; there is an increased mortality rate in the African American population when compared to the white population [12]. For the pediatric population, no significant change in age, race, or sex was seen (Table 1).

There was a consistent trend in both adult and pediatric encounters of fewer motor-vehicle auto injuries during COVID-19 than pre-COVID-19. In adult encounters, when comparing direct numbers of encounters during COVID-19 with those of pre-COVID-19, there were fewer motor-vehicle auto injuries (301 vs. 368) and more other-vehicle injuries (57 vs. 18). For pediatric encounters, there were fewer motor-vehicle auto injuries (11 vs. 22), but more other-vehicle injuries (19 vs. 8). Fewer motor vehicle injuries may have occurred during COVID-19 due to fewer people driving and hence there were fewer chances of motor vehicle collisions. In a study by Yasin et al., a review of global traffic patterns, a large decrease was observed in road traffic and road deaths during COVID-19 [13]. They showed a decrease in trauma admissions from road traffic accidents due to empty lanes and less crowding. There were more other-vehicle injuries, which could include bikes and scooters, the modes of transport people may have been relying more on during the COVID-19 period. With fewer group activities and more outdoor activities encouraged, the sales of bikes and scooters steadily increased throughout the pandemic, resulting in a higher proportion of injuries being seen from this mechanism [14].

Interestingly, fall encounters increased during COVID-19. The impact that COVID-19 has had on the elderly population not only included detrimental health effects, but societal effects including increased isolation leading to higher rates of depression, anxiety, and dementia [15]. We hypothesize that the incidents of falls increased among the elderly population due to the decreased availability of professional caregivers during COVID-19. With fewer family members and professional help available, elderly patients may have been more susceptible to falling.

Gunshot injuries increased significantly among adult traumas (78 during pre-COVID-19 vs. 120 during COVID-19, p=0.01), but there were not enough pediatric gunshot encounters for comparisons to be drawn. Gun sales hit record highs shortly after the spread of COVID-19 and the number of firearms sold in the period of March-August doubled in 2020 compared to the same period in 2019 [16]. Pediatric firearm injuries remain a serious issue in the United States and a further increase in pediatric firearm injuries during COVID-19 was seen [17]. Our results align with other studies detailing this increase in firearm ownership and injuries during COVID-19.

With a change in injury type and mechanism of injury, it was thought that there would be a change in procedures performed as well. There was a significant difference in the number of ORIFs that were carried out, with more being performed during COVID-19 than pre-COVID-19 (235 vs. 178), but no difference was seen related to open reduction external fixation. Interestingly, more ORIFs occurred during COVID-19, which runs counter to recent articles detailing a trend towards non-operative treatment if possible and delaying elective treatments [18]. Due to the nature of many orthopedic trauma injuries, non-operative treatment may not have been feasible. During COVID-19, there were more fractures of the femur, forearm, lower leg and ankle, and shoulder and upper arm. Even though there were increased numbers of ORIFs during COVID-19, there is a possibility that more fractures presented as open, as evidenced in Figure 2. Further research is needed to characterize the severity of these fractures to elucidate why more ORIFs were carried out during COVID-19.

There was a significant change in the number of non-accidental trauma encounters, with more occurring during pre-COVID-19 than during COVID-19 among both adult (15 vs. 11) and pediatric (29 vs. 9) populations. There is a high suspicion about individuals being subjected to increased neglect and abuse due to increased time spent at home during COVID-19 [19]. As for fewer non-accidental trauma encounters seen during COVID-19, concerns have been raised whether this was due to fewer patients being seen for these traumas, rather than fewer traumas of this type occurring. Time spent at activities away from home, where non-accidental trauma could be identified and reported, considerably decreased during the pandemic.

The main limitation of our study is the inconsistency in the reporting of procedures, injury, intent, and mechanism of injury. This lack of consistent reporting can make statistical analysis and the generalizability of our results challenging. There were very few pediatric patient encounters reported during both periods (171 vs. 182), leading to particular difficulty in statistical comparisons for the pediatric population. For adult patients, it was difficult to draw statistical comparisons between injuries that were rarer and had fewer reported numbers. For these injuries, a larger time period is needed for a robust analysis but is unfeasible due to the COVID-19 pandemic’s shifting impact on the healthcare community. Another major limitation of our study was that our data is not representative of the general population and our conclusions are limited to patients with orthopedic injuries. Because orthopedic injuries and trauma injuries do not always overlap with non-accidental injuries, suicides, or domestic violence, there are patients that we may not have been able to include in our study. We saw a decrease in non-accidental trauma, but that may be due to orthopedic surgery or trauma surgery not being consulted for many of those cases. Further research that looks specifically into each category regardless of whether services were consulted or not is needed to gain deeper insights into the topic.

## Conclusions

Overall, our study found no significant change in the number of encounters during COVID-19 as compared to the year directly preceding the pandemic; however, we did find a significant increase in gunshot injuries and other-vehicle injuries, and a significant decrease in motor-vehicle injuries. Of particular interest were the increases in fall injuries and the change in non-accidental injuries that were encountered during the pandemic. Our study outlines a possible trend in injury patterns seen in our institution that can be correlated with the COVID-19 pandemic and the lockdown period. This information is useful for the healthcare system since it demonstrates that resources may not need to be harbored in preparation for a significant increase or decrease in orthopedic trauma from a pandemic or lockdown period. As a level I facility, resources including supplies and staffing need to be allocated to and continued to be provided for emergency rooms and operative services throughout a pandemic. This study demonstrates the need for resources to remain available for surgical specialties throughout a pandemic as incidents of trauma do not decrease but actually seem to increase. Further research that aims to compare more time periods and further stratifying variables needs to be conducted for a better understanding of the topic.
